# Performance Simulation and Fused Filament Fabrication Modeling of the Wave-Absorbing Structure of Conductive Multi-Walled Carbon Nanotube/Polyamide 12 Composite

**DOI:** 10.3390/polym15040804

**Published:** 2023-02-05

**Authors:** Baigang Han, Yan Wang

**Affiliations:** Material Science and Engineering School, Wuhan Institute of Technology, Wuhan 430074, China

**Keywords:** fused filament fabrication (FFF), polymer-matrix composites (PMCs), electrical properties, electromagnetic wave absorption, nanostructures

## Abstract

Fused filament fabrication (FFF) is a reliable method for fabricating structured electromagnetic wave (EMW) absorbers from absorbing materials. In this study, polymer-matrix composites were prepared using polyamide 12 (PA12) which was recovered from selective laser sintering (SLS) as the substrate and multi-walled carbon nanotubes (MWCNT) as the filler. The CST software is used for simulation calculation and study of electromagnetic wave absorption characteristics of composite materials. After that, based on the obtained parameters and results, modeling was carried out, and finally, EMW absorbers with various microstructures were fabricated by FFF. For the honeycomb structure sample, when the side length is 5 mm and the height is 2 mm, the minimum return loss (RL) of the composite at 15.81 GHz is −14.69 dB, and the maximum effective absorption bandwidth is 1.93 GHz. These values are consistent with the simulation results. The pyramid structure has better absorbing performance than plate structure and honeycomb structure. According to simulation calculations, the pyramid structure shows the best performance at an angle of 28°. The absorption performance of the printed pyramid structure sections exceeded the simulated values, with effective absorption bandwidth (EAB) reaching all frequencies from 2 to 18 GHz, with a minimum return loss of −47.22 dB at 8.24 GHz.

## 1. Introduction

The widespread application of electronic products has brought great convenience to people’s lives, but it has also produced electromagnetic radiation, which has become the fourth largest source of pollution after water, air, and noise [1,2,3]. Therefore, the development of high-performance absorbing materials and absorbing structures has become increasingly urgent in military and civilian applications  [4,5].

Recently, the development of radar-absorbing material (RAM) with better processability has become very valuable and popular. Adding wave absorbers into the polymer matrix is considered to be an efficient and feasible way to fabricate RAM [6,7,8]. A large number of studies have shown that multi-walled carbon nanotubes (MWCNTs), as excellent fillers that can improve the mechanical strength, electrical properties, and wave absorbing properties of composites, are widely used as wave absorbers. At the same time, their cost is relatively low, and they have great practical application value [9,10,11,12]. However, their compatibility with the polymeric matrix as a wave absorber is a very acute problem. If the compatibility between the two is poor, it greatly affects the comprehensive properties of the composite material [13]. Therefore, many researchers use different modifiers to modify MWCNTs to improve their compatibility [14,15,16], and they have also tried to use different ratios of MWCNTs, hoping to get the best ratio parameters. 

At the same time, the use of materials with certain wave absorption properties to manufacture certain structures can significantly further improve their wave absorption properties. In particular, structural EWAMs have attracted widespread attention not only because they can be used to adjust the impedance matching of the absorber and enhance absorption performance but also because of their excellent mechanical properties. The structural design of RAM has been proven by many experiments to effectively reduce its RL and expand its effective absorption bandwidth (EAB; RL < 10 dB, EMW energy absorption > 90%) [17,18,19].

However, limited by traditional processing methods, the design and manufacture of structural EMW absorbers have been greatly affected  [20,21]. It is difficult and expensive to realize the free design of various structures of structural EWAM through traditional manufacturing systems. The emergence of three-dimensional (3D) printing technology has broadened the design of absorbing structures, to obtain a better absorbing effect [22,23]. The excellent productivity and flexibility of FFF have greatly been used to develop modern structural materials for applications in aerospace, automotive industries, and medical engineering. Structural absorbers have good electromagnetic absorption properties, can function as mechanical bearings, and can be used as structural components such as aircraft wings and pelvic fins  [24,25,26]. At present, the main absorbers include laminated, sandwich, tapered, and honeycomb structures [27,28,29].

The design of absorbers usually considers two aspects: (1) good impedance-matching characteristics at the interface to reduce the reflection of electromagnetic waves and ensure that the electromagnetic waves can enter the absorbing material as much as possible; and (2) good EMW attenuation characteristics, from which the incident EMW can be converted into Joule heat energy to the greatest extent in the absorbing material and dissipated. In general, there have been some explorations of 3D printing structural absorbers. 3D printing can realize the rapid manufacture of 3D complex structural absorbers. Using the characteristics of layered manufacturing, a gradient change of the impedance of each layer of materials can be achieved. Therefore, 3D-printed structural absorbers have good application prospects. However, there are few types of absorbing materials that can be successfully applied to printing. On the other hand, there are less explorations on printing absorbing structures.

Therefore, this study aimed to fill the recovered PA12 in SLS with MWCNTs to provide a functional material with good mechanical properties and strong EMW absorption for FFF. The simulation of structural wave absorption was then performed using their electromagnetic parameters, which were further tested and confirmed by 3D printing. In addition, the filling of MWCNT improves the performance of recovered PA12 in FFF, which plays a role in environmental protection and savings while overcoming the internal stress of PA12 itself, which can improve its warpage during the printing process. Herein, MWCNT/PA12 composites were prepared via the wet-wetting process in combination with mechanical ball milling and melt extrusion.

## 2. Experimental Section

### 2.1. Materials

Recycled polyamide 12 (PA12) powder from selective laser sintering (SLS) was provided by Huake Sanwei Technology Co., Ltd.(Wuhan, China); multi-walled carbon nanotubes (MWCNTs) were purchased from Zhongsen Linghang Technology Co., Ltd. (Shenzhen, China); dodium dodecyl benzene sulfonate(SDBS) was provided by Sinopharm Chemical Reagents Co., Ltd. (Shanghai, China). The purity of SDBS is greater than or equal to 88%. The type of PA12 is the same as VESTAMID type. The basic parameters of MWCNT are displayed in Table 1.

### 2.2. Preparation of MWCNT/PA12 Composites

**Figure 1:**
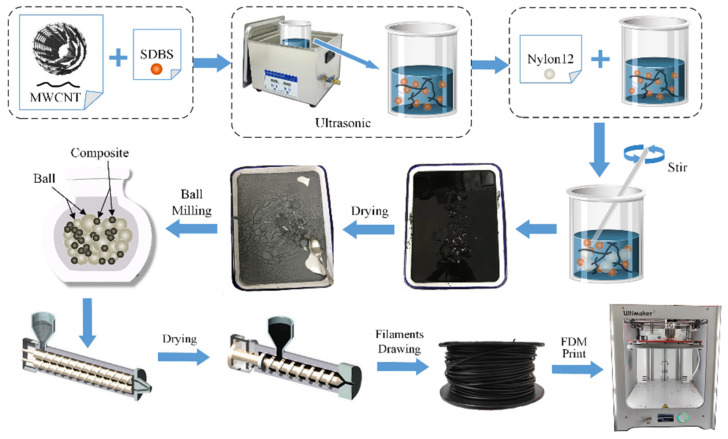
MWCNT/PA12 composites preparation process.

MWCNTs were treated with sodium dodecylbenzene sulfonate (SDBS) as a modifier to improve their compatibility with PA12. First, SDBS and MWCNTs were dissolved in deionized water, and the solution was sonicated for 8 h to disperse the agglomerated MWCNTs. The dried PA12 powder was then added to the MWCNT-SDBS solution and blended by mechanical stirring. Finally, the mixed solution was dried to obtain MWCNT/PA12 powder, which was further blended by ball milling and melt extrusion to obtain 10 wt.% MWCNT/PA12 composite pellets. The overall preparation process is shown in Figure 1. The properties of the resulting 10 wt.% MWCNT/PA12 composites are listed in Table 2.

### 2.3. Preparation of the MWCNT/PA12 Filaments

The MWCNT/PA12 composite filaments were prepared with a single-screw extruder (TP-07, Songhu Plastic Machinery, Dongguan, China). The nozzle diameter of the single screw extruder was 2.85 mm. The processing temperature was 180, 235, 215 °C, and the cooling water temperature was 50 °C with host speed of 1440 rpm and tractor speed of 350 rpm.

### 2.4. Progress of FFF Printing

**Figure 2:**
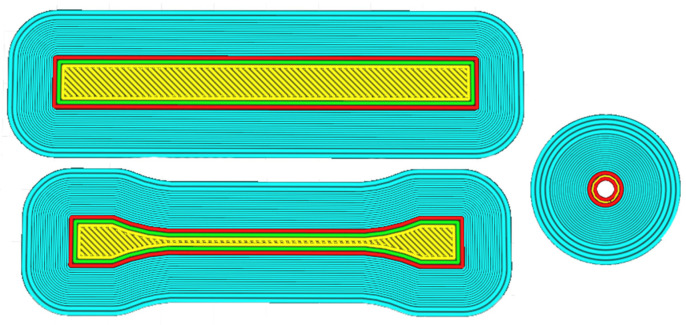
Printing path of straight, dumbbell, and concentric annular-shaped samples.

The equipment used for FFF printing was an FFF 3D printer (Ultimaker 3, Ultimaker, Utrecht, The Netherlands). The X-direction was chosen as the build direction of the printed samples. The print path is shown in Figure 2. For the setting of printing process, thickness was 0.1 mm; speed was 30 mm/s; temperature was 245 °C; and diameter was 0.4 mm.

### 2.5. Characterization and Testing

The tensile properties of the dumbbell-shaped samples were determined using a universal testing machine (5960, INSTRON, Boston, MA, USA) according to GB/T16421-1996 with a load speed of 20 mm/min. The flexural properties were determined using the universal testing machine according to GBT9341-2008 at a strain rate of 2 mm/min. Impact strength tests were performed using a cantilever beam impact testing machine (XJU-22, Chengde Experimental Machine Co., Ltd., Chengde, China) according to GBT1843-2008 at a pendulum energy of 5.5 J.

The morphologies of the MWCNT/PA12 were observed by scanning electron microscope (SEM, JSM-5510 LV, Japan Electron Optics Laboratory Co., Ltd., Tokyo, Japan). The electromagnetic parameters of the 10 wt.% MWCNT/PA12 composite was measured using a vector network analyzer (N5224A, Keysight Technologies, Santa Rosa, CA, USA) in the frequency of 0.3–18 GHz.

**Figure 3:**
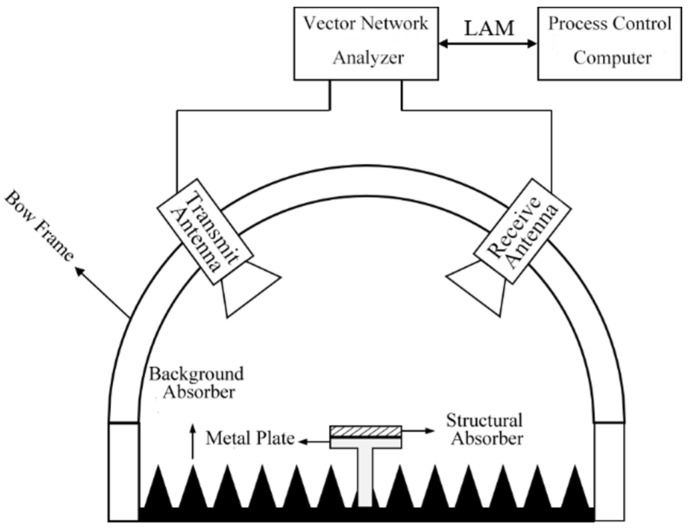
Schematic of arc reflection test system.

In this study, the radar wave reflectivity of the structural absorber was tested in the frequency of 2–18 GHz by the bow method using the vector network analyzer, according to the GJB2038A-2011 standard. As shown in Figure 3, the structural absorber is placed on a metal plate in an anechoic chamber in which the experimental temperature is 23 °C and the relative humidity is 70%.

## 3. Results and Discussion

### 3.1. Effect of MWCNT Content on Properties of MWCNT/PA12 Composites

After the PA12 powder used for SLS is sintered at high temperatures many times, reactions such as crosslinking and degradation occur between the molecules, which increases the molecular weight gap and reduces the performance. It is not enough to continue to support the use of SLS, and it is generally scrapped. Therefore, PA12 recovered from SLS was combined with MWCNT, and the effects of different mass fractions of MWCNT on the various properties of MWCNT/PA12 composites were explored.

With the increase in the mass fraction of MWCNT, the volume resistivity of the composite decreased greatly, the mechanical properties showed a trend of first increasing and then decreasing and processability gradually decreased. When the mass fraction of MWCNT reached 7% and 10%, the resistivity of the composite decreased by 11 and 13 orders of magnitude compared with pure PA12, respectively, and further increasing the proportional resistivity of MWCNTs does not decrease the resistivity much. Considering various properties, 10%MWCNT/PA12 composite material can be considered to have the best comprehensive properties. Therefore, this composite material was selected for experiments such as printing and simulation. The parameter comparison is shown in Table 3.

### 3.2. Appearance and Microscopic Morphology of the Printed Samples

**Figure 4:**
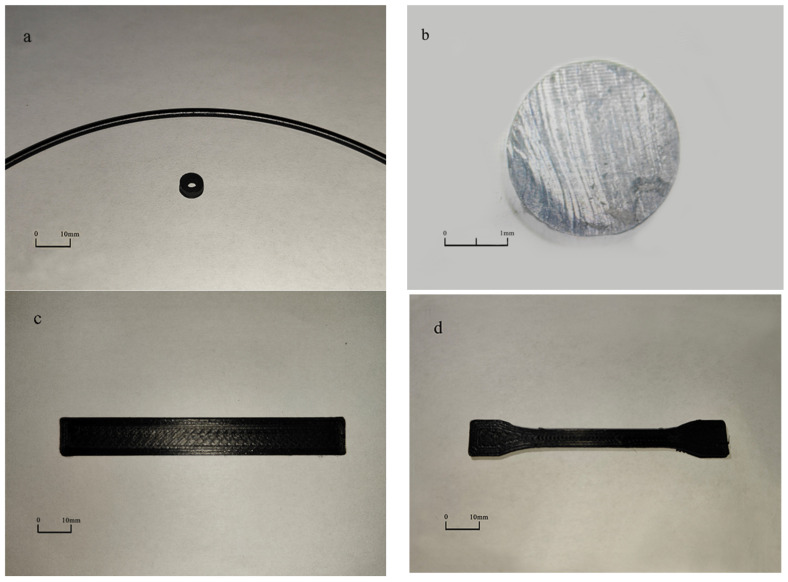
10 wt.% MWCNT/PA12 composite filament and samples. (**a**) Macro-morphology of filament and printed concentric annular-shaped sample. (**b**) Cross-section of filament. (**c**) Printed straight sample. (**d**) Printed dumbbell sample.

Figure 4 shows a filament for FFF prepared using the 10 wt.% MWCNT/PA12 composite and samples printed using the filament. As shown in Figure 4a,b, the filament has a good appearance, the surface has a smooth texture and no defects, and its cross-section shows a dense state without pore defects. This shows that this is an excellent composite material that can be used for FFF.

**Figure 5:**
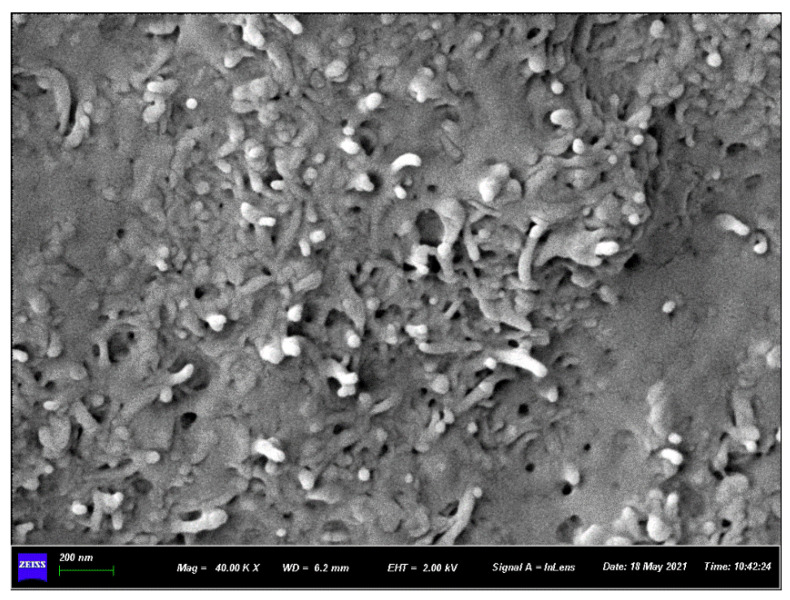
Impact profiles of 10 wt.% MWCNT/PA12 composite samples.

The SEM image in Figure 5 is obtained by observing the impact section of the MWCNT/PA12 composite. The MWCNT is well dispersed, and there is essentially no agglomeration. This results in better strengthening and toughening in the PA12 and smaller volume resistivity of the composites.

### 3.3. Influence of Printing Process Parameters on the Performance

The printing process parameters have a large impact on the sample performance. Therefore, it is meaningful to compare the effects of different temperatures and speeds on the mechanical properties and electrical conductivity of the samples. According to the results, the optimal printing process parameters can be obtained.

**Figure 6:**
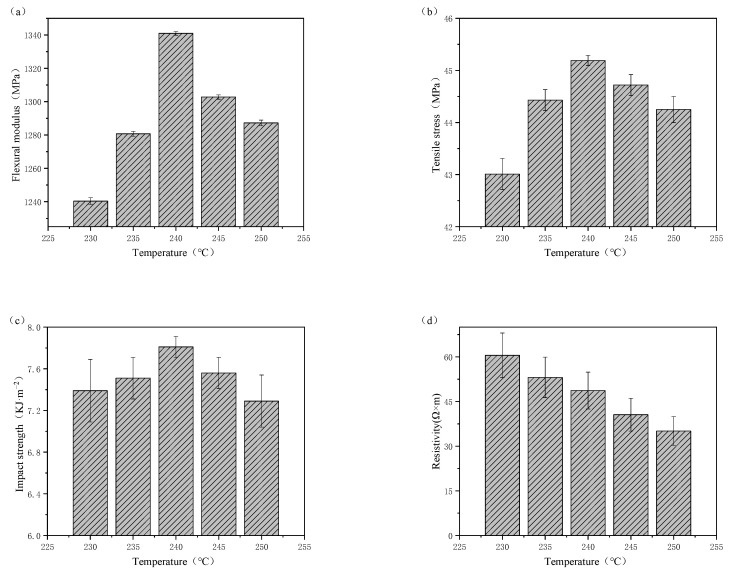
Effect of printing temperature on the (**a**) flexural modulus, (**b**) tensile strength, (**c**) impact strength, and (**d**) volume resistivity of printed samples.

Figure 6 shows the effect of printing temperature on the properties of printed samples. The mechanical properties of the printed samples first increase and then decrease with increasing printing temperature. When the printing temperature was 240 °C, the flexural modulus, tensile strength, and impact strength of the printed samples were the highest. Lower temperatures can lead to incomplete melting and poor flow, and higher temperatures can prevent the material from cooling in time to deposit, both of which result in performance degradation. The resistivities of the MWCNT/PA12 composites gradually decreased with increasing printing temperature. This is because as the printing temperature rises, and the gap between layers decreases, which is more conducive to the passage of current. Their resistivities were lower by 14 orders of magnitude than that of pure PA12.

**Figure 7:**
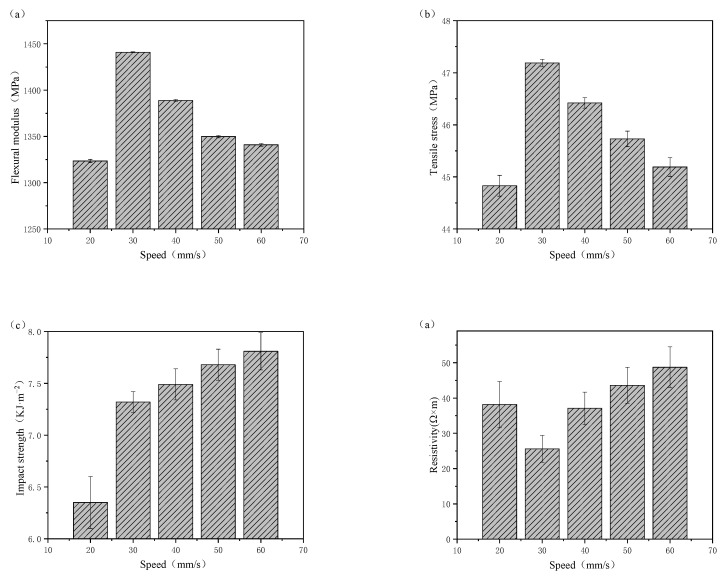
Effect of printing speed on the (**a**) flexural modulus, (**b**) tensile strength, (**c**) impact strength, and (**d**) volume resistivity of printed samples.

Figure 7 shows the effect of printing speed on the properties of printed samples. With increasing printing speed, the flexural modulus and tensile strength of the printed samples first increased and then decreased, and the impact strength increased continuously. The best comprehensive properties of the composites were obtained at a printing speed of 30 mm/s. Inadequate printing speed led to too much extruded and bonded filament, and the nozzle could not move away in time, which eventually made the printing of each layer too thick and uneven, which affects the bonding between layers. When the printing speed was 30 mm/s, the volume resistivity of the printed samples was the lowest and the mechanical properties were good. Its resistivity was lower by 14 orders of magnitude than that of pure PA12. With the further increase in the printing speed, excessive speed led to the reduction of printing quality, the materials could not be completely bonded, and the bonding between layers became worse, which further reduced the flexural modulus of the material.

### 3.4. Analysis of Electromagnetic Properties

In general, the dielectric dissipation factor (tan δ_e_ = ε″/ε′) and the magnetic dissipation factor (tan δ_m_ = μ″/μ′) are used to describe the ability of materials to dissipate electromagnetic waves. For an absorber, these parameters (ε′, ε″, μ′, μ″, tan δ_e_ and tan δ_m_) affect its performance together.

**Figure 8:**
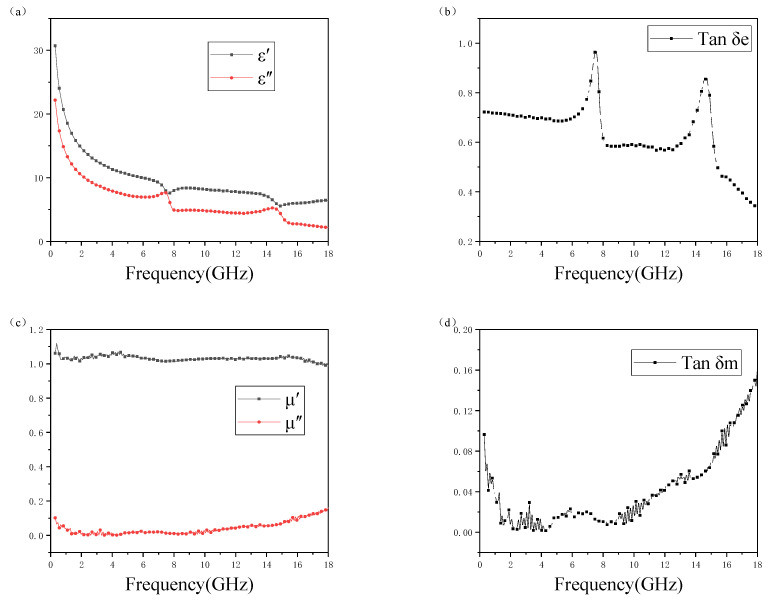
Electromagnetic properties of composites. (**a**) Real part (ε′) and imaginary part (ε″). (**b**) Dielectric loss tangent Tan δ_e_. (**c**) Real part (μ′) and imaginary part (μ″). (**d**) Magnetic loss tangent Tan δ_m_.

The real part εʹ gradually decreased with increasing frequency and fluctuated in the range of 6.46–30.70, which is a typical dispersion behavior caused by high-frequency polarization delay; ε″ also decreased with frequency, oscillating in the 2.16–22.17 range. It is worth noting that there is a large ratio throughout the frequency band, so the total dissipation factor is large. As shown in Figure 8, the composites exhibited higher dielectric loss tangents in the C-band and Ku-band. Due to their favorable conductance, composite materials usually have high dielectric loss factors.

It can be observed from Figure 8c,d that the values of μ′, μ″, and tan δ_m_ fluctuate in the ranges of 0.98–1.12, 0–0.16, and 0–0.16, respectively. Such values were presented because the composite is not magnetic.

### 3.5. Simulation and Analysis of Electromagnetic Wave Plate Absorption Performance

**Figure 9:**
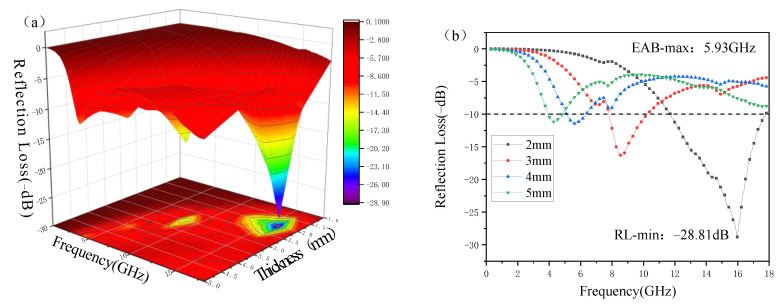
(**a**) 3D theoretically calculated reflection loss (RL) spectra of MWCNT/PA12 composites and (**b**) 2D RL plots.

Figure 9 shows the frequency dependence of the RLs of 10 wt.% MWCNT/PA12 samples as their thicknesses are increased from 1 mm to 5 mm. When the thickness is 2 mm, it exhibits a significantly enhanced EMW-absorbing performance, with a lower RL-min of −28.81 dB at 16.00 GHz and a higher EAB-max of 5.93 GHz (Figure 6b), which suggests excellent EMW absorption performance. This can be due to the composite having proper impedance matching and being capable of drastically dissipating electromagnetic waves. The two key factors that determine the electromagnetic wave absorption characteristics are impedance matching and damping constant.

### 3.6. Simulation and Experimental Verification of Honeycomb Structural Absorber

As one of the most classic structures in nature, the honeycomb structure is also widely used in the design and preparation of absorbers. The adjustable parameters of the honeycomb structural absorber include the size of the honeycomb aperture, the height of the structure, the electromagnetic parameters of the absorbing coating, the thickness of the absorbing coating, etc.; additionally, the design freedom is high.

**Figure 10:**
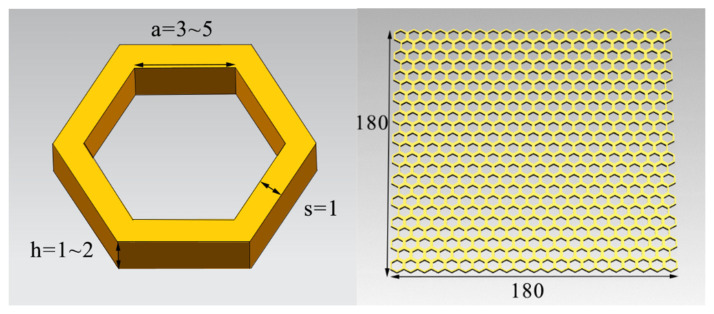
Parameters of the honeycomb structure (unit: mm).

The honeycomb structural absorber was modeled and simulated using CST software, and the size of the honeycomb structure with the best absorbing performance was selected. The simulated honeycomb size parameters are shown in Figure 10, where the inner side length of the hexagon of a single honeycomb unit is ‘a’, the thickness is ‘s’, and the honeycomb height is ‘h’ (mm). The overall size of the honeycomb structure is a square of dimensions 180 mm × 180 mm.

**Figure 11:**
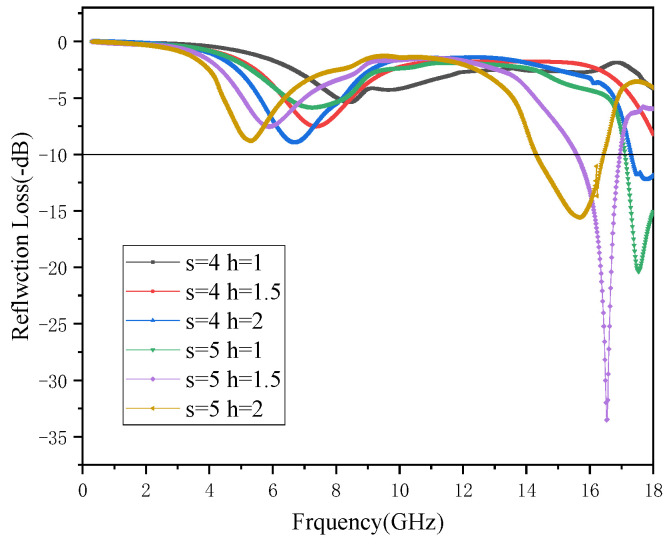
RL spectra of different honeycomb models.

Figure 11 shows the relationship between the microwave-absorbing properties of the composites and the honeycomb size. The figure shows the change of RL in the frequency range of 0.3–18 GHz when ‘a’ increases from 3 mm to 5 mm, ‘h’ increases from 1 mm to 2 mm, and ‘s’ is 1 mm.

It can be seen from the figure that when ‘a’ is 4–5 mm and ‘h’ is 1–2 mm, the honeycomb structure exhibits a certain wave-absorbing performance, and when “a” is 5 mm, its absorption is better. When “a” is 5 mm and “h” is 1 mm, 1.5 mm and 2 mm, the composite obtained the minimum RL at 17.54 GHz, 16.53 GHz and 15.68 GHz, respectively. Their minimum RLs were −20.35 dB, −33.52 dB, and −15.57 dB. Their maximum effective absorption bandwidths were 0.85 GHz, 1.01 GHz, and 2.12 GHz, respectively. It can be seen that with the increase in “h”, the minimum absorption peaks of composite materials were shifted to lower frequencies, and their maximum effective absorption bandwidths were also increased. Among them, ‘a’ = 5 mm, ‘h’ = 2 mm was a better parameter; it had a smaller RL and a larger absorption frequency.

**Figure 12:**
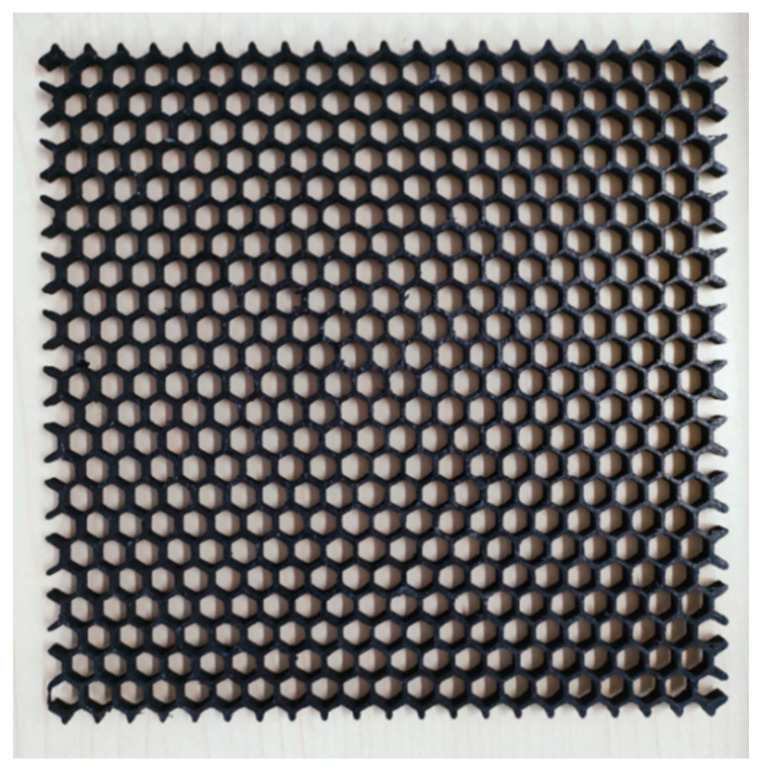
The printed honeycomb structural absorber.

Therefore, the size parameters of ‘a’ = 5 mm, ‘s’ = 1 mm, and ‘h’ = 2 mm were selected as the honeycomb unit, and the overall size of the honeycomb structural absorber was 180 mm × 180 mm × 2 mm; it was printed by FFF, as shown in Figure 12.

**Figure 13:**
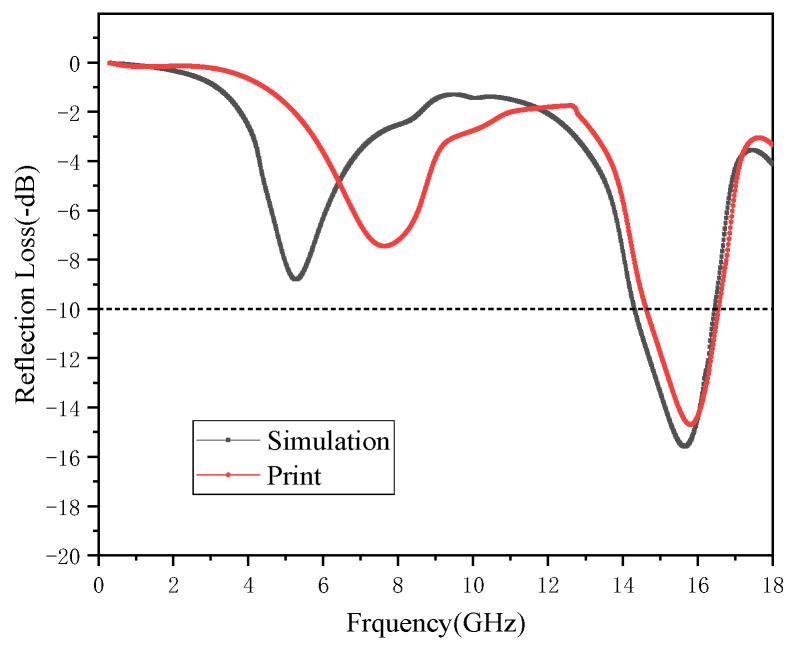
RL spectra of the printed honeycomb absorber and simulated honeycomb model.

**Figure 14:**
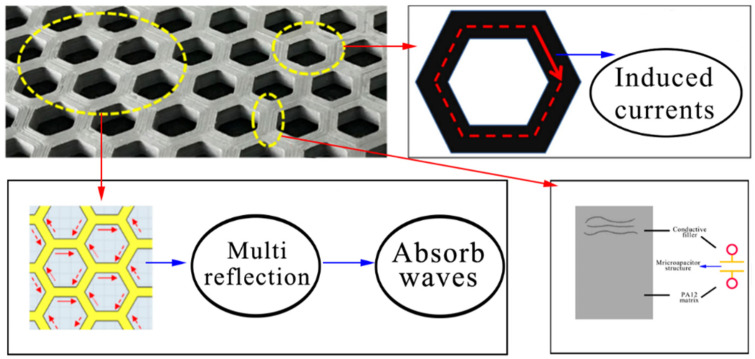
The absorbing principle of the honeycomb absorber.

Figure 13 shows a comparison between the absorbing performance of the printed honeycomb model and the simulated honeycomb model. The overall loss variation of both was similar, and they had the same absorption peak. The composites achieve the minimum RL of −15.81 dB at 14.69 GHz, and the maximum effective absorption bandwidth is 1.93 GHz. Its absorbing principle is shown in Figure 14. The honeycomb structure produces regular-shaped voids inside the material, which increases the area of the internal interface of the material. When the electromagnetic wave enters the structure, a large number of reflection effects are generated at these internal interfaces, which causes further losses in the electromagnetic wave in a large amount.

However, the performance of the printed part is slightly worse than the wave absorption in the simulation. This is because the size of the single honeycomb unit of the honeycomb model was small, which led to a decrease in accuracy, and the PA12 crystallization also affected the accuracy. Improving the parameters and conditions of printing or optimizing the size parameters of the absorbing structure can further improve its absorbing performance.

### 3.7. Simulation and Experimental Verification of the Pyramid-Structural Absorber

**Figure 15:**
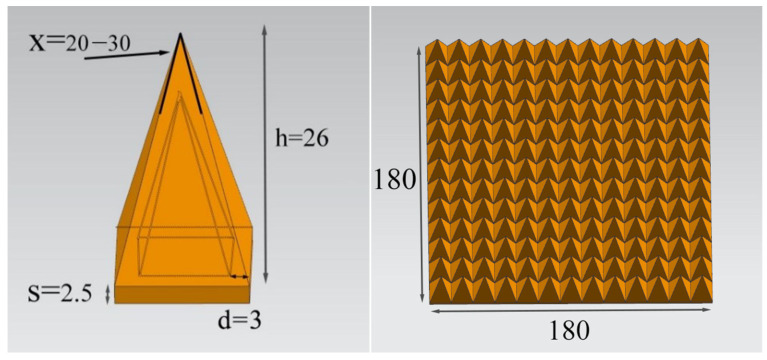
Parameters of the pyramid structure (unit: mm).

The pyramid-structural absorber was modeled and simulated using CST software, and the wave absorption of different angles of the pyramid was compared. The structural parameters of the simulation are shown in Figure 15. The bottom thickness of a single pyramid element is 2.5 mm, the height of the pyramid is 26 mm, the wall thickness of the structure is 3 mm, and the included angle is ‘X’ (°); the overall dimensions of the printed pyramid are 180 mm × 180 mm square.

**Figure 16:**
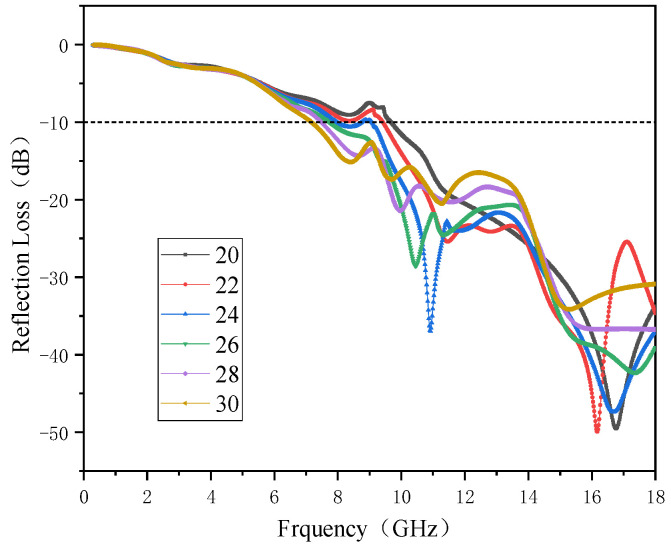
RL spectra of pyramid models with different angles.

**Figure 17:**
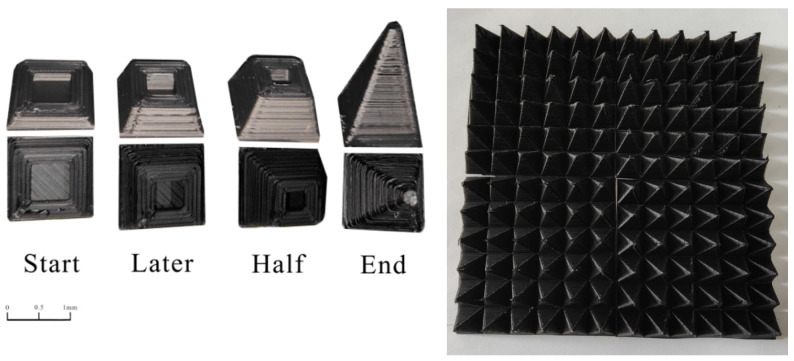
Printed pyramid structural absorber.

Figure 16 shows the change in the absorbing performance of the composites with the angle of the pyramid. The figure shows the change of RL in the frequency range of 0.3–18 GHz when the angle of a single pyramid element increases from 20° to 30°. The absorbing structures with different angles show a similar trend, and the absorbing bandwidth increases continuously with an increase in the angle. When the angle is a minimum of 20°, the pyramid structure achieved a minimum RL value of −49.53 dB at 16.76 GHz, and a maximum effective absorption bandwidth of 8.32 GHz. When the angle increased to 28°, the minimum RL value of the pyramid structure was −36.72 dB, which was obtained at 16.18 GHz, and its maximum effective absorption bandwidth was 10.51 GHz. When the angle was the maximum 30°, the pyramid structure obtained the minimum RL value of 31.98 dB at 16.46 GHz, and the maximum effective absorption bandwidth was 10.85 GHz. A pyramid structural absorber with an angle of 28° was printed by FFF. The process of printing and the printing structure of 180 mm × 180 mm × 26 mm are shown in Figure 17.

**Figure 18:**
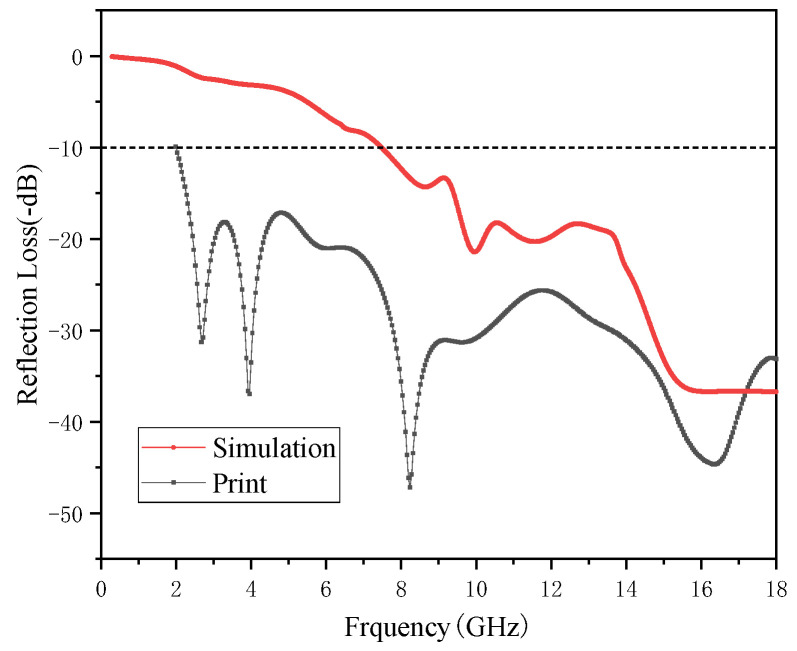
RL spectra of the printed pyramid absorber and simulating pyramid model.

**Figure 19:**
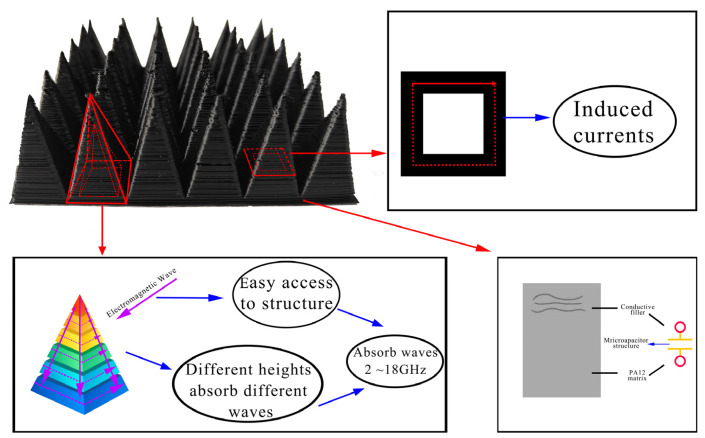
The absorbing principle of the pyramid absorber.

Figure 18 shows a comparison between the absorbing performance of the printed pyramid and the simulated pyramid model. The simulated and actual printed pyramids have similar absorption peaks, and the change trends are essentially the same. However, the absorbing performance of the printed part exceeds the analogue value, so all frequencies from 2 to 18 GHz can absorb waves. The minimum RL of −47.22 dB is obtained at 8.24 GHz. Its absorbing principle is shown in Figure 19. It can be known from the flat plate model that as the thickness increases, the absorption peak shifts to the high-frequency band, while the absorption of the low-frequency band generally requires a lower thickness. In the pyramid structure, due to the characteristics of the processing method, the bonding between the layers is not tight enough, and the effect is similar to the superposition of multiple thin layers so that the electromagnetic waves in the low-frequency band can be better absorbed, and this also may be because the printed pyramid absorber has appropriate surface roughness. When the printing material was extruded from the nozzle, the MWCNT had a certain orientation, which was beneficial for the absorption of electromagnetic waves. Thus, composites can have proper impedance matching and strong attenuation. Electromagnetic waves of different bands can easily enter and be lost. The pyramid wave absorber obtained by printing exhibited excellent wave absorption performance and has great application potential.

## 4. Conclusions

The effects of different printing temperatures and speeds on the material properties were compared, and the optimal printing process parameters were obtained. The optimal printing process parameters for printing with MWCNT/PA12 composite material were the printing temperature of 240 °C and the printing speed of 30 mm/s, and the printed samples have the best comprehensive performance at this time. The absorbing properties of different structures were explored by simulation and printing. For the plate-structural sample, the MWCNT/PA12 composite with a thickness of 2 mm has the best comprehensive absorbing performance. A minimum RL value of −28.81 dB was achieved at 16.00 GHz, while a maximum effective absorption bandwidth of 5.93 GHz was also obtained. For the honeycomb structural sample, the absorbing performance of the honeycomb structure was simulated using CST software. When the side length was 5mm and the height was 2 mm, the electromagnetic absorption performance of the composite material was the best. Its minimum RL value was −15.57 dB at 15.68 GHz, while the maximum effective absorption bandwidth was 2.12 GHz. The composite material obtained a minimum RL value of −14.69 dB at 15.81 GHz and a maximum effective absorption bandwidth of 1.93 GHz. In addition, it was demonstrated that the performance of the printed part was close to the wave absorption in the simulation. Similarly, for the pyramid-structural sample, the absorbing performance of the pyramid structure was also simulated by CST software. When the angle was 28°, the structure had the lowest RL and the highest absorbing bandwidth. The simulated and actual printed pyramids had similar absorption peaks, and the change trends were essentially the same. However, the absorbing performance of the printed part exceeded the analogue value, the EBS reached all frequencies from 2 to 18 GHz, and the minimum RL −47.22 dB was obtained at 8.24 GHz. Thus, the pyramid wave absorber has great application potential.

## Figures and Tables

**Table 1:** Basic parameters of multi-walled carbon nanotubes.

Basic Parameters	Length (μm)	Diameter (nm)	Density (g/cm^3^)	Purity (%)
MWCNT	10–30	5–15	0.06	>97

**Table 2:** Properties of 10 wt.% MWCNT/PA12 composites.

Property	Test Method	Unit	Value
Melt index	GB/T3682-2000	g/10 min	8.64
Vicat	GB/T1633-2000	°C	162.6
Flexural modulus	GBT9341-2008	MPa	1369.22
Tensile strength	GB/T16421-1996	MPa	52.08
Impact strength	GBT1843-2008	KJ·m^−2^	8.56
Volume resistivity	Megger	Ω·m	46.2

**Table 3:** Properties of composites with different proportions of MWCNTs.

MWCNT (wt.%)	Tensile Strength (MPa)	Flexural Modulus (MPa)	Impact Strength (KJ·m^−2^)	Volume Resistivity (Ω·m)	Vicat (°C)	Melt Index (g/10 min)
Pure PA12	43.82 ± 1.96	1022.05 ± 36.68	3.57 ± 0.11	10^13^~10^14^	138.7 ± 0.51	47.2 ± 1.27
3	49.05 ± 1.29	1180.77 ± 35.11	12.27 ± 0.27	1.62 × 10^6^ ± 2.29 × 10^3^	148.5 ± 0.36	27.1 ± 1.05
5	54.2 ± 0.24	1297.53 ± 31.16	9.64 ± 0.19	3.92 × 10^5^ ± 159	152.8 ± 0.21	22.79 ± 0.56
7	53.06 ± 0.21	1351.71 ± 38.87	8.8 ± 0.15	4.83 × 10^3^ ± 25.71	157.8 ± 0.19	20.58 ± 0.31
10	52.08 ± 0.18	1369.22 ± 43.22	8.56 ± 0.21	46.2 ± 3.87	161.3 ± 0.25	8.64 ± 0.11
12	46.92 ± 1.53	1371.41 ± 41.58	7.22 ± 0.14	26.9 ± 2.76	163.4 ± 0.28	3.71 ± 0.05

## Data Availability

Data is unavailable due to privacy; no new data were created.
